# Supplementary material for: Integrating artificial intelligence (AI) in healthcare: advancing older adults’ health management in Saudi Arabia through AI-powered chatbots
Source: PeerJ Comput Sci. 2025 Mar 27;11:e2773. doi: 10.7717/peerj-cs.2773 (PMC12190517; doi:10.7717/peerj-cs.2773)
Supplement: Supplemental Information 2 [file peerj-cs-11-2773-s002.docx]

**Appendix A**. Measurement of research variables

| **Construct** | **Items** | | **Reference** |
| --- | --- | --- | --- |
| Perceived hedonic motivation (HED) | HED1 | Using healthcare chatbot application is fun. | Iancu & Iancu (2023) ; O’Brien (2010) |
|  | HED2 | Using healthcare chatbot application is pleasant |  |
|  | HED3 | Using healthcare chatbot application is enjoyable and fascinating |  |
| Facilitating Condition (FAC) | FAC1 | I have the resources necessary to use the healthcare chatbot applications. | Park et al. (2011); Venkatesh et al (2003); Venkatesh et al.(2012) |
|  | FAC2 | I have the knowledge necessary to use healthcare chatbot applications. |  |
|  | FAC3 | My computer is compatible with the healthcare chatbot applications. |  |
|  | FAC4 | A specific group is available for assistance with difficulties associated with the healthcare chatbot applications. |  |
| Social Influence (SOC) | SOC1 | People who are important to me think that I should use healthcare chatbot applications for my healthcare services. | Venkatesh et al (2003); Venkatesh et al.(2012) |
|  | SOC2 | People who influence my behaviors recommend that I use healthcare chatbot applications |  |
| Performance Expectancy (PER) | PER1 | The healthcare chatbot gives me the right answers. | Balakrishnan et al. (2022) ; ,Venkatesh et al (2003); Venkatesh et al.(2012) |
|  | PER2 | The healthcare chatbot understands complex conversation. |  |
|  | PER3 | The healthcare chatbot performance is equal to humans. |  |
| Effort Expectancy (EFF) | EFF1 | The healthcare chatbot is easy to use. | Slade et al. (2015) ; Venkatesh et al.(2012) |
|  | EFF2 | Learning to use healthcare chatbot is easy. |  |
| Confirmation (CON) | CON1 | My experience with using the healthcare chatbot application was better than I expected | Tam et al. (2020) ; Lee (2010) |
|  | CON2 | The service level provided by the healthcare chatbot application was better than I expected |  |
|  | CON3 | The healthcare chatbot applications can meet demands in excess of what I required for the service. |  |
|  | CON4 | Overall, most of my expectations from using the healthcare chatbot applications were confirmed. |  |
| Satisfaction (SAT) | SAT1 | I am satisfied with my decision to use healthcare chatbot. | Bhattacherjee (2001a, 2001b). |
|  | SAT2 | My decision to use the healthcare chatbot application was a wise one. |  |
|  | SAT3 | I am happy with my earlier decision to use healthcare chatbot application |  |
| Continuous intention to use AI-Powered Chatbots (COT) | COT1 | I intend to continue using healthcare chatbot application rather than discontinue its use. | Al-Sharafi et al. (2023); Thong et al. (2006) |
|  | COT2 | I intend to continue using healthcare chatbot applications rather than other alternative means. |  |
|  | COT3 | If I could, I would like to sustain my use of healthcare chatbot applications for my healthcare services. |  |
| Perceived humanness (HPU) | HPU1 | I perceive that this chatbot is friendly to me during the conversation | Hu et al. (2021); Go & Sundar (2019); Dziergwa et al. (2018) |
|  | HPU2 | The chatbot gave some smart suggestions based on my responses. |  |
|  | HPU3 | I feel this chatbot is sociable |  |
| Perceived Security (SEC) | SEC1 | I feel secure in providing sensitive information to chatbot. | Noor et al.  (2022) |
|  | SEC2 | The information that chatbot has about me is protected. |  |
|  | SEC3 | I trust that my personal information with chatbot application will not be misused. |  |
