# Supplementary material for: Integrating artificial intelligence (AI) in healthcare: advancing older adults’ health management in Saudi Arabia through AI-powered chatbots
Source: PeerJ Comput Sci. 2025 Mar 27;11:e2773. doi: 10.7717/peerj-cs.2773 (PMC12190517; doi:10.7717/peerj-cs.2773)
Supplement: Supplemental Information 3 [file peerj-cs-11-2773-s003.pdf]

Integrating AI in Healthcare: Advancing Older Adults' Health Management through AI-powered chatbots

Dear participants

We are conducting research that aims to explore the determinants of sustained use of AI-powered healthcare chatbots among older adults.

The research will be conducted using questionnaire. Participants who are 60 and above will be asked to respond to a series of questions related to healthcare chatbots applications. It is estimated that the completion of the questionnaire will take approximately 15 minutes.

By participating in this research study, you acknowledge that:

1. You have read and understood the purpose and methods of the research study as explained above.
2. You consent to voluntarily participate in this study and you have the right to withdraw at any time without providing a reason.
3. You acknowledge that your responses will remain anonymous, confidential, and will be used solely for research purposes.
4. You understand that your participation involves no known physical, psychological, or social risks.
5. You agree to answer the questionnaire to the best of your ability and provide honest and accurate responses.
6. You consent to the use of your answers for the purpose of publication or presentation in scientific conferences, provided that no personally identifiable information will be disclosed.

If you have any questions or concerns about this research study, you may contact Dr. Sabah Al-Somali by e-mail.

Please check (X) if you confirm that you are 60 years old or older and voluntarily agree to participate in this study.

☐ I confirm that I voluntarily agree to participate in this study with a clear understanding of the purpose and methods involved.

Thank you in advance for your time!

Cordially
Dr. Sabah Abdullah Al-Somali
MIS department, King Abdulaziz University
e-mail: saalsomali@kau.edu.sa

Part 1: General Information

Please tick (✓) the appropriate box for each question.

1. **Gender**

□ Male	□ Female

2. ***Education level***

□ *High school*	□ *Bachelor*
□ *Master*	□ *Doctorate*

3. ***Income***

□ *Prefer not to say*	□ *Less than 16,000 SAR*
□ *Between 16,000 SAR and 26,000 SAR*	□ *More than 26,000 SAR*
□ *Do Not Know*	

4. **Reasons for using Healthcare Chatbot**

□ To schedule appointments	□ To get prescription refill
□ To get medical Assistant	□ To get information about disease prevention
□ To inquiry about insurance claims	

5. **Healthcare Chabot Access**

□ From PC	□ From Smart Phone/Tablet

Part 2: AI-powered healthcare chatbots Solutions

This section looks for your thoughts and current practices regarding the use of AI-powered healthcare chatbots solutions. **Please circle the number which best describes your opinion.**

1. The following question is asked to ascertain your opinion (or expectations) about the motivations to use AI-powered healthcare chatbots solutions. **Please circle the scale closest to your views**

Strongly Disagree	Disagree	Partially	Agree	Strongly Agree
**1**	**2**	**3**	**4**	**5**

1. Using healthcare chatbot application is fun.	1	2	3	4	5
2. Using healthcare chatbot application is pleasant	1	2	3	4	5
3. Using healthcare chatbot application is enjoyable and fascinating	1	2	3	4	5

2. The following question is asked to ascertain your opinion (or expectations) about the resources you have to use AI-powered chatbots solutions. **Please circle the scale closest to your views**

Strongly Disagree	Disagree	Partially	Agree	Strongly Agree
**1**	**2**	**3**	**4**	**5**

1. I have the resources necessary to use the healthcare chatbot applications.	1	2	3	4	5
2. I have the knowledge necessary to use healthcare chatbot applications.	1	2	3	4	5
3. My computer is compatible with the healthcare chatbot applications.	1	2	3	4	5
4. A specific group is available for assistance with difficulties associated with the healthcare chatbot applications.	1	2	3	4	5

3. The following question is asked to ascertain your opinion (or expectations) about the social influence to use AI-powered healthcare chatbots solutions. **Please circle the scale closest to your views**

Strongly Disagree 1	Disagree 2	Partially 3	Agree 4	Strongly Agree 5

1. People who are important to me think that I should use healthcare chatbot applications for my healthcare services.	1	2	3	4	5
2. People who influence my behaviors recommend that I use healthcare chatbot applications	1	2	3	4	5

4. The following question is asked to ascertain your opinion (or expectations) about the performance of AI-powered healthcare chatbots solutions. **Please circle the scale closest to your views**

Strongly Disagree 1	Disagree 2	Partially 3	Agree 4	Strongly Agree 5

1. The healthcare chatbot gives me the right answers.	1	2	3	4	5
2. The healthcare chatbot understands complex conversation.	1	2	3	4	5
3. The healthcare chatbot performance is equal to humans.	1	2	3	4	5

5. The following question is asked to give your opinion (or expectations) about how easy it is to use AI-powered healthcare chatbots solutions. **Please circle the scale closest to your views.**

Strongly Disagree 1	Disagree 2	Neutral 3	Agree 4	Strongly Agree 5

1. The healthcare chatbot is easy to use.	1	2	3	4	5
2. Learning to use healthcare chatbot is easy.	1	2	3	4	5

6. The following question is asked to give your opinion (or expectations) about your satisfaction with AI-powered healthcare chatbots solutions. **Please circle the scale closest to your views.**

Strongly Disagree 1	Disagree 2	Partially 3	Agree 4	Strongly Agree 5

1. I am satisfied with my decision to use healthcare chatbot.	1	2	3	4	5
2. My decision to use the healthcare chatbot application was a wise one.	1	2	3	4	5
3. I am happy with my earlier decision to use the healthcare chatbot application	1	2	3	4	5

7. The following question is asked to give your opinion (or expectations) about your experience using AI powered healthcare chatbots solutions. **Please circle the scale closest to your views.**

Strongly Disagree 1	Disagree 2	Partially 3	Agree 4	Strongly Agree 5

1. My experience with using the healthcare chatbot application was better than I expected	1	2	3	4	5
2. The service level provided by the healthcare chatbot application was better than I expected	1	2	3	4	5
3. The healthcare chatbot applications can meet the demands in excess of what I required for the service.	1	2	3	4	5
4. Overall, most of my expectations from using the healthcare chatbot applications were confirmed.	1	2	3	4	5

5. The following question is asked to look for your intention to continue using AI powered healthcare chatbots solutions. **<u>Please circle the scale closest to your views.</u>**

Strongly Disagree<br>1	Disagree<br>2	Partially<br>3	Agree<br>4	Strongly Agree<br>5

1. I intend to continue using healthcare chatbot application rather than discontinue its use.	1	2	3	4	5
2. I intend to continue using healthcare chatbot applications rather than other alternative means.	1	2	3	4	5
3. If I could, I would like to sustain my use of healthcare chatbot applications for my healthcare services.	1	2	3	4	5

6. The following question is asked to give your opinion about how chatbots reply to your inquiries. **<u>Please circle the scale closest to your views.</u>**

Strongly Disagree<br>1	Disagree<br>2	Partially<br>3	Agree<br>4	Strongly Agree<br>5

1. I perceive that this chatbot is friendly to me during the conversation	1	2	3	4	5
2. The chatbot gave some smart suggestions based on my responses.	1	2	3	4	5
3. I feel this chatbot is sociable	1	2	3	4	5

7. The following question is asked to give your opinion about the security of AI powered healthcare chatbots solutions. **<u>Please circle the scale closest to your views.</u>**

Strongly Disagree<br>1	Disagree<br>2	Partially<br>3	Agree<br>4	Strongly Agree<br>5

1. I feel secure in providing sensitive information to chatbot.	1	2	3	4	5
2. The information that chatbot has about me is protected.	1	2	3	4	5
3. I trust that my personal information with chatbot application will not be misused.	1	2	3	4	5

***** Thank you for completing this questionnaire, your contribution is greatly appreciated*****
